# Efficacy and Safety of DNV3 (a Lymphocyte‐activation Gene 3–blocking Antibody) Combined With Toripalimab and Chemotherapy in Advanced Melanoma: An Open‐label, Single‐arm Clinical Trial

**DOI:** 10.1002/mco2.70648

**Published:** 2026-03-02

**Authors:** Jing Lin, Lizhu Chen, Ling Chen, Dingyi Wang, Yuping Lu, Huishan Zhang, Ping Chen, Wei Yan, Zuoxiang Xiao, Yu Chen

**Affiliations:** ^1^ Department of Medical Oncology Clinical Oncology School of Fujian Medical University Fujian Cancer Hospital, NHC Key Laboratory of Cancer Metabolism Fuzhou China; ^2^ Cancer Bio‐Immunotherapy Center Clinical Oncology School of Fujian Medical University Fujian Cancer Hospital Fuzhou China; ^3^ Department of Phase I Clinical Trial Ward Clinical Oncology School of Fujian Medical University Fujian Cancer Hospital Fuzhou China; ^4^ Department of Clinical Research Center Zhejiang Shimai Pharmaceutical Co., Ltd Hangzhou Zhejiang Province China

**Keywords:** LAG‐3 inhibitor, PD‐1 refractory melanoma, mucosal melanoma, chemo‐immunotherapy, toripalimab

## Abstract

Despite the remarkable therapeutic advances achieved with immune checkpoint inhibitors in advanced melanoma, treatment options remain limited for patients with refractory subtypes. This study evaluated a novel combination of DNV3 (anti‐LAG‐3), toripalimab (anti‐PD‐1), and chemotherapy (nab‐paclitaxel/cisplatin) in 27 Asian patients with unresectable or metastatic melanoma (77.8% [21/27] previously treated with anti‐PD‐[L]1 and 22.2% [6/27] treatment‐naïve mucosal melanoma; subtypes: 13 mucosal, 6 acral, 5 cutaneous, and 3 of unknown primary origin). The regimen achieved an overall response rate (ORR) of 44.4%, which was further elevated to 54.5% in the subgroup of 11 patients with hepatic metastases. Notably, it also demonstrated substantial efficacy in anti‐PD‐(L)1‐resistant cases, with a 42.9% ORR and a median progression‐free survival (PFS) of 7.36 months. Among treatment‐naïve mucosal melanoma, the ORR reached 50%. At data cutoff, median overall survival remained unreached in all cohorts. Grade ≥3 treatment‐related adverse events initially occurred in 55.6% of participants; subsequent dose modification of nab‐paclitaxel (from 260 mg/m^2^ to 200 mg/m^2^) improved tolerability, reducing the incidence of grade ≥3 events to 22.2%. Immune‐related toxicities (grade 3–4, 22.2%) were clinically manageable. Therefore, the combination of LAG‐3/PD‐1 blockade and chemotherapy demonstrated promising efficacy, notably in treatment‐naïve mucosal melanoma with liver metastases. (Chinese Clinical Trial Registry number, ChiCTR2400079543)

## Graphical abstract

This investigator‐initiated trial demonstrates that LAG‐3/PD‐1 dual blockade (DNV3 + toripalimab) combined with chemotherapy achieves a 44.4% overall response rate (ORR) in advanced melanoma, showing particular efficacy in anti‐PD‐(L)1‐resistant patients (42.9% ORR, 7.36‐month median PFS), treatment‐naïve mucosal melanoma (50.0% ORR), and those with liver metastases (54.5% ORR). Following dose optimization, the regimen exhibited improved tolerability, supporting this quadruplet approach as a promising therapeutic strategy for refractory melanoma populations with urgent unmet needs, warranting further validation in larger cohorts.

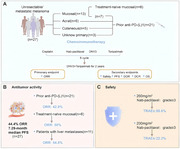

## 1 Introduction

Melanoma remains one of the most aggressive forms of skin cancer, responsible for nearly 90% of skin cancer–related deaths despite comprising only 4% of cutaneous malignancies [1]. Although its incidence is highest among Caucasian populations (23.3 per 100,000 in Sweden) [2], outcomes for advanced disease remain unfavorable, with a 5‐year overall survival rate of merely 30% [3]. The introduction of immune checkpoint inhibitors (ICIs) targeting CTLA‐4 and programmed cell death‐1 (PD‐1) has reshaped therapeutic strategies [4, 5]. Notably, the combination of nivolumab(a PD1‐ blocking antibody) and relatlimab (a Lymphocyte‐activation gene 3(LAG 3)–blocking antibody) demonstrated superior clinical benefit in the RELATIVITY‐047 trial, with an objective response rate (ORR) of 43.1% compared with 32.6% for nivolumab monotherapy, and a lower incidence of grade 3–4 adverse events (AEs: 21.1% vs 55.0% for ipilimumab–nivolumab) [6, 7], leading to FDA approval in 2022.

Therapeutic outcomes, however, remain unsatisfactory for melanoma subtypes predominant in Asian populations. Mucosal melanoma, the most frequent subtype in Asia [8, 9], exhibits limited responsiveness to immunotherapy, with ORRs ranging from 10% to 40% even under dual CTLA‐4/PD‐1 blockade [10]. Combining ICIs with chemotherapy has emerged as a potential strategy to overcome such resistance. Paclitaxel derivatives, including nab‐paclitaxel, are capable of inducing immunogenic cell death [11, 12, 13, 14], and early investigations have reported synergistic interactions with PD‐1 inhibitors, resulting in a substantial ORR increase from 20% to 75% in acral melanoma [15].

DNV3 (a LAG 3–blocking antibody), a novel humanized anti‐LAG‐3 monoclonal antibody developed by Shima Medicine, remains without a commercial designation. Although structurally similar to the approved agent relatlimab as an IgG4 monoclonal antibody, DNV3 differs markedly in the Fv region sequences, resulting in distinct binding behaviors and biological activities. Relatlimab primarily recognizes LAG‐3–expressing T cells with limited affinity for LAG‐3–positive B cells, whereas DNV3 exhibits high‐affinity binding to both cell types, particularly T cells. Functionally, DNV3 effectively inhibits the interactions between LAG‐3 and its two principal ligands, MHC‐II and FGL1, while relatlimab efficiently disrupts only the LAG‐3–MHC‐II interaction with minimal interference in LAG‐3–FGL1 binding. These molecular and functional disparities suggest that DNV3 may confer broader or more potent immunoregulatory activity within certain tumor microenvironments.

Based on this mechanistic foundation, a therapeutic regimen combining DNV3 with toripalimab (PD‐1 inhibitor) and chemotherapy (nab‐paclitaxel/cisplatin) was investigated. This triple‐targeted approach aims to overcome therapeutic resistance in refractory melanoma by concurrently modulating LAG‐3/PD‐1 immune checkpoints, enhancing chemotherapy‐induced immunogenicity, and exerting direct cytotoxic effects on tumor cells.

## 2 Results

### 2.1 Patient Characteristics

**FIGURE 1:**
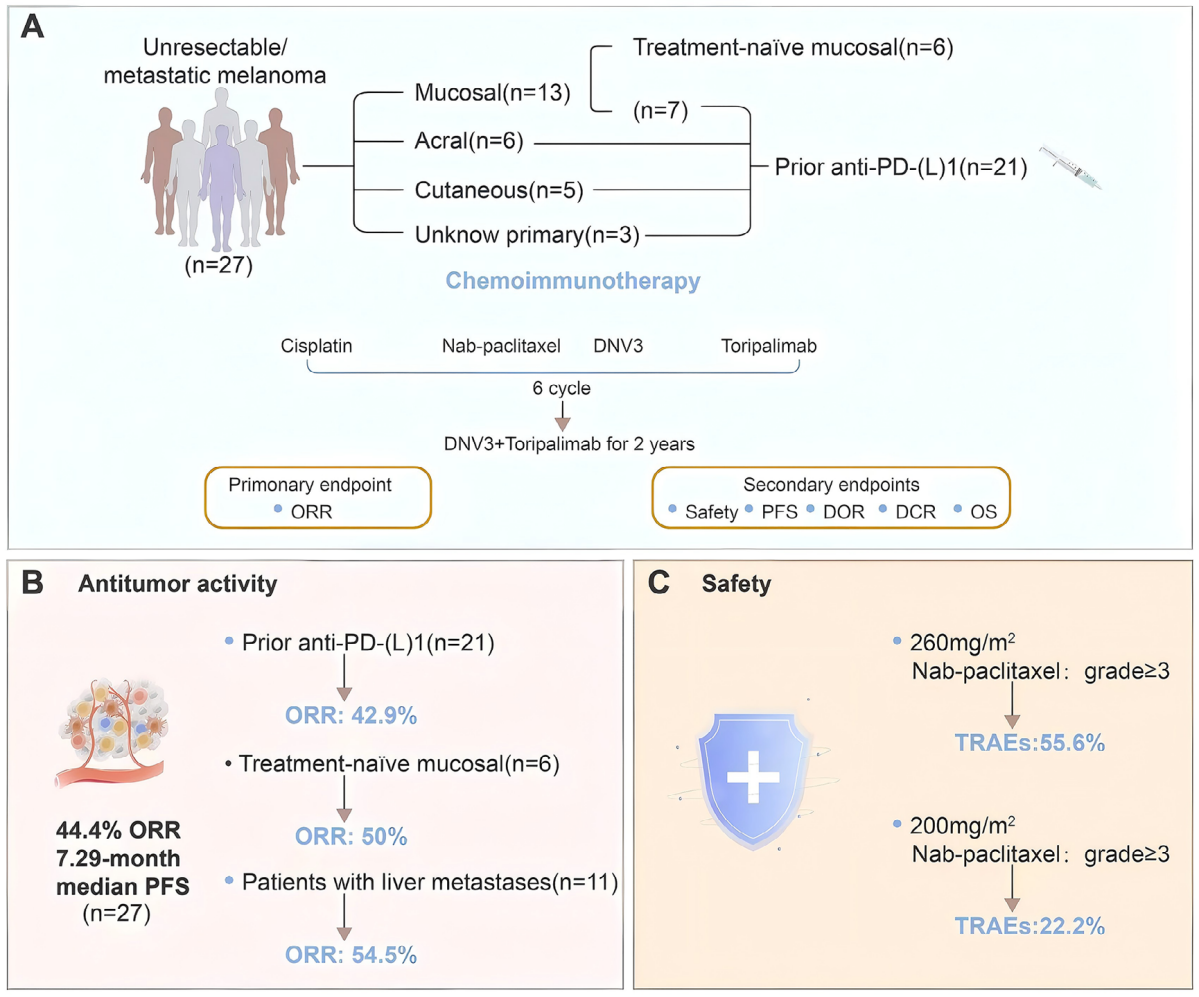
Study overview. (A) Treatment regimen, assessment schedule, study framework, and analytical population. (B) Antitumor efficacy metrics. (C) Safety summary. TRAEs, treatment‐related adverse events; ORR, objective response rate; PFS, progression‐free survival; PD‐(L)1, programmed death‐(ligand)1; DOR, duration of response; DCR, disease control rate; OS, overall survival.

This study aimed to assess the efficacy and safety of DNV3 plus toripalimab and chemotherapy in an Asian cohort with advanced melanoma (Figure 1A‐C). Between February and August 2024, a total of 27 Asian patients were enrolled (median age 59 years; 48.1% male). All participants exhibited Eastern Cooperative Oncology Group (ECOG) 0–1 performance status, with prior exposure to anti‐PD‐(L)1 therapy in 21 cases and six presenting with treatment‐naïve mucosal melanoma. Baseline features included LDH >2×ULN in 3 patients, PD‐L1 CPS ≥5 in 5, BRAF mutations in 4, and metastatic involvement of the liver and brain in 11 and 2 cases, respectively (Table 1). At a median follow‐up of 8.5 months (IQR 3.1–10.0), the median treatment duration was 7.2 months (IQR 2.3–8.6). Data were censored on April 8, 2025.

**TABLE 1:** Summary of Demographic Characteristics.

	Total (N = 27) n (%)
Age (years)	
Nx (missing)	27 (0)
Mean (SD)	57.4 (11.58)
Median (Q1, Q3)	59.0 (50.0, 68.0)
Range	34 ‐ 72
Sex[n(%)]	
Male	13 (48.1)
Female	14 (51.9)
ECOG score[n(%)]	
0	2 (7.4)
1	25 (92.6)
LDH (U/L)	
Nx (missing)	27 (0)
Mean (SD)	384.6 (472.36)
Median (Q1, Q3)	250.0 (182.0, 322.0)
Range	136 ‐ 2423
LDH[n(%)]	
≤ULN	14 (51.9)
ULN<x≤2*ULN	10 (37.0)
>2*ULN	3 (11.1)
Melanoma subtype [n(%)]	
Mucosal	13 (48.1)
Cutaneous	5 (18.5)
Acral	6 (22.2)
Unknown	3 (11.1)
Disease stage and M status [n(%)]	
IV,M1a	7 (25.9)
IV,M1b	3 (11.1)
IV,M1c	15 (55.6)
IV,M1d	2 (7.4)
Liver metastases [n(%)]	
Yes	11 (40.7)
No	16 (59.3)
Brain metastases [n(%)]	
Yes	2 (7.4)
No	25 (92.6)
BRAF[n(%)]	
Positive	4 (14.8)
Negative	23 (85.2)
Unknown	0
PD‐L1 status [n(%)]	
<1	14 (51.9)
≥1	10 (37.0)
Unknown	3 (11.1)
PD‐L1 status [n(%)]	
<5	19 (70.4)
≥5	5 (18.5)
Unknown	3 (11.1)
Prior anti‐PD‐(L)1 therapy [n(%)]	
Yes	21 (77.8)
No	6 (22.2)
Prior systemic regimens [n(%)]	
0	6 (22.2)
1	18 (66.7)
2	2 (7.4)
3	1 (3.7)
Baseline Target Lesion Size (mm)	
Nx (missing)	27 (0)
Mean (SD)	72.35 (44.884)
Median (Q1, Q3)	70.60 (31.60, 100.60)
Range	10.1 ‐ 183.7
Treatment time (months)	
Nx (missing)	27 (0)
Mean (SD)	4.34 (2.348)
Median (Q1, Q3)	4.20 (2.30, 5.60)
Range	0.7 ‐ 9.4
Follow‐up duration (months)	
Nx (missing)	27 (0)
Mean (SD)	5.31 (2.436)
Median (Q1, Q3)	5.50 (3.10, 7.00)
Range	1.2 ‐ 10.9

*Note*: N = number of subjects in the analysis population, Nx = number of subjects with non‐missing value, n = number of subjects in the specific category, percentage (%) = (n/N)*100.

Treatment time = (the actual duration of therapy from the first to the last dose, last date of drug administration—C1D1 of drug administration+1)/30.4375. Follow‐up duration = (last date of followup time—C1D1 of drug administration+1)/30.4375.

Abbreviations: ECOG, eastern cooperative oncology group; LDH, lactate dehydrogenase; PD‐L1, programmed death ligand 1; SD, standard deviation.

### 2.2 Antitumor Activity

**FIGURE 2:**
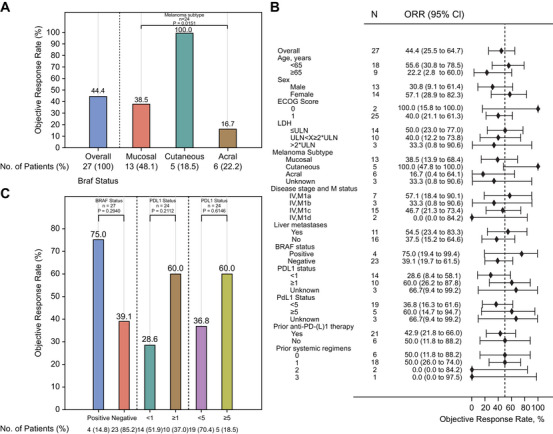
Treatment response characteristics. (A) ORR across melanoma subtypes—mucosal (n = 13), cutaneous (n = 5), and acral (n = 6)—evaluated per RECIST v1.1. (B) Forest plot depicting ORR variability among clinical subgroups defined by treatment history and metastatic status. (C) Predictive biomarker assessment: ORR categorized by BRAF mutation (positive/negative) and PD‐L1 expression (≥1% vs <1% tumor proportion score; ≥5% vs <5% tumor proportion score). PD‐L1, programmed death‐(ligand)1; ORR, objective response rate; CI, confidence interval; ECOG, eastern cooperative oncology group; LDH, lactate dehydrogenase.

According to Response Evaluation Criteria in Solid Tumours (RECIST) 1.1 criteria, the overall cohort (n = 27) demonstrated an ORR of 44.4% (95% confidence interval (CI), 25.5–64.7) (Figure 2A). Response analysis across subtypes revealed distinct outcomes: 100% for cutaneous, 38.5% for mucosal, and 16.7% for acral melanoma (Figure 2A). Factors associated with higher response rates included age <65 years, ECOG PS 0, LDH ≤ ULN, M1a classification, liver metastases, and prior exposure to anti‐PD‐(L)1 agents (Figure 2B). Patients previously treated with anti‐PD‐(L)1 (n = 21) achieved an ORR of 42.9% (95% CI, 21.8–66.0) (Table S1), with response rates by subtype of 28.6% for mucosal, 100% for cutaneous, 16.7% for acral, and 33.3% for unknown primary melanoma (Table S1). In treatment‐naïve mucosal melanoma (n = 6), the ORR reached 50.0% (95% CI, 11.8–88.2) (Table S2). Patients with liver metastases exhibited particularly favorable activity (ORR 54.5%, 95% CI, 23.4–83.3; Figure 2B). Among all cases, four carried BRAF mutations while twenty‐three were wild‐type. Next‐generation sequencing (NGS) identified the V600E variant in all BRAF‐mutated patients, with no V600D or other non‐V600 variants detected. BRAF mutation status did not show a statistically meaningful predictive association (mutant: 75% [3/4] vs wild‐type: 39.1% [9/23]; Figure 2C).

**FIGURE 3:**
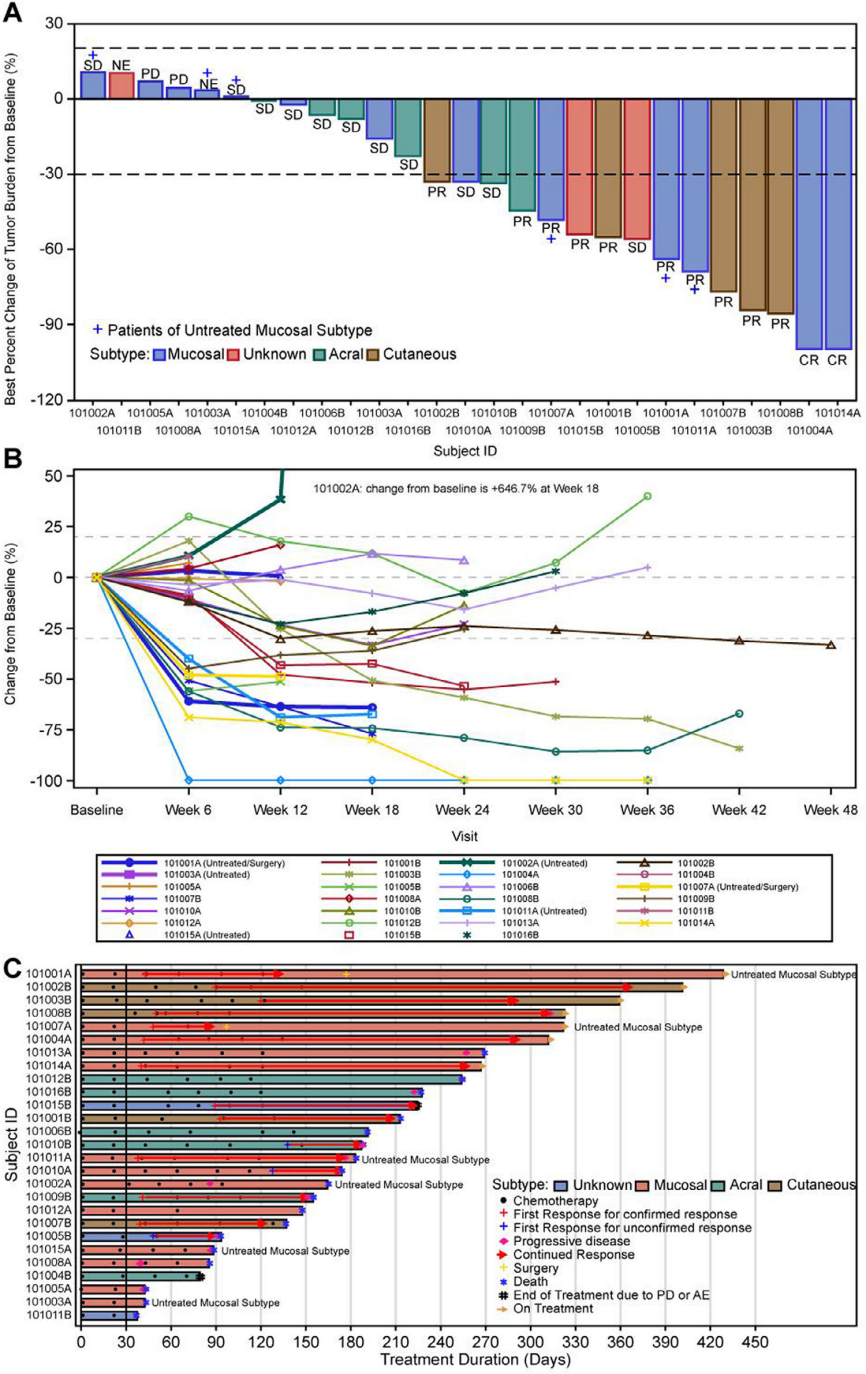
Dynamics of treatment response in advanced melanoma (N = 27). (A) Best overall response per RECIST v1.1 (investigator‐assessed). Waterfall plot depicting maximum tumor reduction from baseline; negative values indicate lesion shrinkage. (B) Temporal evolution of tumor burden. Spider plot illustrating individual tumor size trajectories relative to baseline. (C) Treatment duration and response persistence. Swimmer plot displaying therapy exposure (bar length) and sustained responses (colored segments) among responders. SD, stable disease; NE, not evaluable; PD, progressive disease; PR, partial response; CR, complete response; AE, adverse event.

Among 27 patients with pathologically confirmed melanoma, 2 achieved adequate tumor regression permitting radical surgery following partial response (PR) to treatment. Based on RECIST v1.1, the overall response distribution included 2 complete responses (CRs, 7.4%), 10 PRs (37.0%), 11 cases of stable disease (SD, 40.7%), 2 progressive diseases (PD, 7.4%), and 2 non‐evaluable cases (NE, 7.4%) (Figure 3A,B). The treatment duration for each case is illustrated in Figure 3C.

**FIGURE 4:**
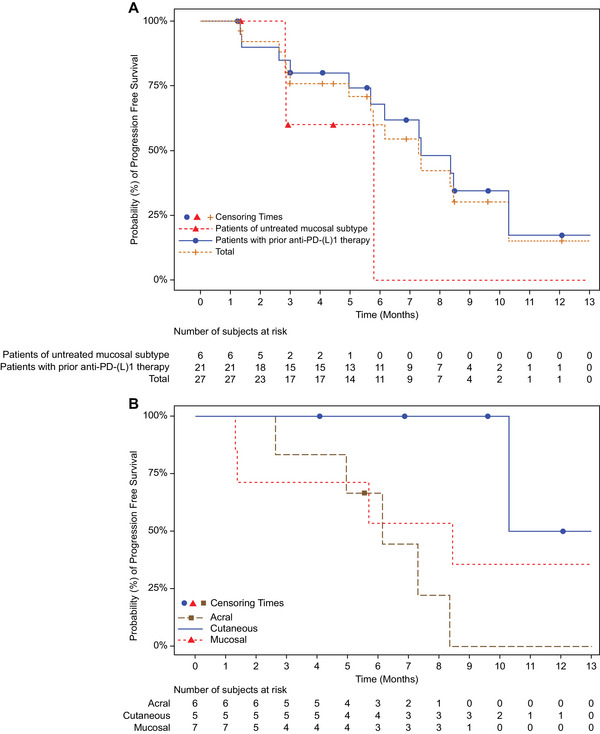
Progression‐free survival analysis. (A) Kaplan–Meier estimates comparing PFS across subgroups: treatment‐naïve mucosal melanoma (n = 6), anti‐PD‐(L)1 pretreated patients (n = 21), and the entire cohort (N = 27). (B) PFS according to melanoma subtype: acral (n = 6), cutaneous (n = 5), and mucosal (n = 13) subgroups. PD‐(L)1, programmed death‐(ligand)1; PFS, progression‐free survival.

The median progression‐free survival (PFS) for the total cohort (n = 27) was 7.29 months (95% CI: 4.96–10.28) (Table S3), while the median overall survival (OS) remained unreached. In patients with liver metastases (n = 11), PFS was 4.96 months (95% CI: 1.38–NE) (Table S3). In patients previously exposed to anti‐PD‐(L)1 therapy (n = 21), PFS was comparable at 7.36 months (95% CI: 4.96–10.28) (Table S4). Conversely, treatment‐naïve mucosal melanoma cases (n = 6) exhibited a median PFS of 5.78 months (95% CI: 2.83–NE) (Table S4). Within the pretreated subgroup, the mucosal subtype demonstrated the longest PFS (8.44 months, 95% CI: 1.31–NE) (Table S5). The 6‐month PFS rate was 60.0% for the entire cohort, 68.1% for pretreated patients, and 40.3% for mucosal cases; corresponding 12‐month rates were 15.1% and 17.2% (Table S6) (Figure 4A). Figure 4B depicts PFS stratified by subtype among the 21 patients previously receiving anti‐PD‐(L)1 therapy. OS rates reached 95.8% at 6 months and 87.8% at 12 months overall, with 100% survival at both time points in treatment‐naïve mucosal melanoma (Table S6).

The median duration of response (DOR) was not achieved in the overall cohort. Patients pretreated with anti‐PD‐(L)1 exhibited a longer median DOR (8.67 months, 95% CI: 3.65–NE) compared with treatment‐naïve mucosal cases (4.57 months) (Table S7). In addition, durable responses persisted in pretreated mucosal and cutaneous subtypes (DOR not reached), whereas acral melanoma showed a shorter DOR of 3.65 months (Table S8).

Analysis of key patient subgroups revealed distinct response patterns to the therapy. Among 13 patients with mucosal melanoma (Table S9), the treatment‐naïve subgroup (n = 6) showed a higher ORR (50%) than the previously treated subgroup (n = 7, ORR 28.6%), with median PFS of 5.78 and 8.44 months, respectively. This trend was accentuated in the liver metastasis cohort (Table S10), where all objective responders (n = 3) were treatment‐naïve mucosal melanoma patients, whereas no response was observed in the previously treated mucosal melanoma patients.

In patients who had received prior anti‐PD‐(L)1 therapy (Table S11), the ORR was 10.0% in cutaneous melanoma (n = 10) and 21.4% in acral melanoma (n = 14), with comparable disease control rates (∼60%).

Furthermore, an exploratory analysis of PD‐L1 expression (Tables S12, S13) showed that patients with cutaneous melanoma had 100% ORR in both CPS≥1 and CPS≥5 subgroups, numerically higher than other subtypes. Across the entire cohort, the ORR was higher in CPS≥1 patients (60.0%) than in CPS<1 patients (28.6%); however, none of these comparisons, including tests for a linear association, reached statistical significance, likely limited by the small sample size of each subgroup.

### 2.3 Treatment‐related Toxicity

The DNV3 combination regimen demonstrated a manageable safety profile. Among the 27 enrolled patients, the initial 18 received nab‐paclitaxel at a dose of 260 mg/m^2^. In this cohort, TRAEs were observed in 94.4% (17/18) of patients, with grade 3–4 TRAEs occurring in 50.0% (9/18), primarily infections (22.2%) and bone marrow suppression (11.1%) (Table 2). Notably, two patients (11.1%) in this group experienced grade ≥3 myelosuppression, one of whom (5.56%) died from a myelosuppression‐associated infection. Based on these safety findings, the dose of nab‐paclitaxel was reduced to 200 mg/m^2^ for the subsequent nine patients. Following this adjustment, the incidence of grade 3–4 TRAEs decreased to 22.2% (2/9), mainly presenting as hematologic events (Table 3). Among these, only one case of grade 3 leukopenia was reported, with no further high‐grade myelosuppressive events observed. Efficacy evaluation revealed a median PFS of 6.1 months in the 260 mg/m^2^ group (n = 18) and 7.3 months in the 200 mg/m^2^ group (n = 9). The reduction in the nab‐paclitaxel dose did not compromise efficacy, as evidenced by comparable PFS outcomes between the dose cohorts.

**TABLE 2:** Summary Treatment‐Related Adverse Events by CTCAE Grade.

Nab‐Paclitaxel dosage(mg/m^2^): 260					
TRAE	Any Grade n (%)	Grade 1–2 n (%)	Grade 3 n (%)	Grade 4 n (%)	Grade 5 n (%)
Treatment‐Related AE	17 (94.4)	7 (38.9)	8 (44.4)	1 (5.6)	1 (5.6)
Anaemia	6 (33.3)	5 (27.8)	1 (5.6)	0	0
Infection	5 (27.8)	0	4 (22.2)	1 (5.6)	0
GGT elevation	4 (22.2)	4 (22.2)	0	0	0
Nausea	4 (22.2)	4 (22.2)	0	0	0
Skin itch	4 (22.2)	4 (22.2)	0	0	0
ALT elevation	3 (16.7)	3 (16.7)	0	0	0
AST elevation	3 (16.7)	3 (16.7)	0	0	0
WBC count decreased	3 (16.7)	3 (16.7)	0	0	0
Blood glucose elevation	2 (11.1)	2 (11.1)	0	0	0
Bone marrow suppression	2 (11.1)	0	1 (5.6)	0	1 (5.6)
Diarrhea	2 (11.1)	2 (11.1)	0	0	0
Hypercholesteremia	2 (11.1)	2 (11.1)	0	0	0
Hypochloridemia	2 (11.1)	2 (11.1)	0	0	0
Hypokalemia	2 (11.1)	2 (11.1)	0	0	0
Hyponatremia	2 (11.1)	1 (5.6)	1 (5.6)	0	0
PLT count decreased	2 (11.1)	2 (11.1)	0	0	0
Proteinuria	2 (11.1)	2 (11.1)	0	0	0
Rash	2 (11.1)	1 (5.6)	1 (5.6)	0	0
ALP elevation	1 (5.6)	1 (5.6)	0	0	0
Abdominal distension	1 (5.6)	1 (5.6)	0	0	0
Acute heart failure	1 (5.6)	0	0	1 (5.6)	0
Arrhythmia	1 (5.6)	0	0	1 (5.6)	0
Belching	1 (5.6)	1 (5.6)	0	0	0
Constipation	1 (5.6)	1 (5.6)	0	0	0
Creatinine elevation	1 (5.6)	1 (5.6)	0	0	0
Dizziness	1 (5.6)	1 (5.6)	0	0	0
Fatigue	1 (5.6)	0	1 (5.6)	0	0
Fever	1 (5.6)	1 (5.6)	0	0	0
Gastrooesophageal burning	1 (5.6)	1 (5.6)	0	0	0
Hiccup	1 (5.6)	1 (5.6)	0	0	0
Hypertriglyceridemia	1 (5.6)	1 (5.6)	0	0	0
Hypoadrenal function	1 (5.6)	0	1 (5.6)	0	0
Hypoalbuminemia	1 (5.6)	1 (5.6)	0	0	0
Leucoderma	1 (5.6)	1 (5.6)	0	0	0
Musculoskeletal pain	1 (5.6)	1 (5.6)	0	0	0
Neutrophil count decreased	1 (5.6)	1 (5.6)	0	0	0
Neutrophil count elevation	1 (5.6)	1 (5.6)	0	0	0
Respiratory failure	1 (5.6)	0	0	1 (5.6)	0
Rhinitis	1 (5.6)	1 (5.6)	0	0	0
Sinus tachycardia	1 (5.6)	1 (5.6)	0	0	0
Total bilirubin elevation	1 (5.6)	1 (5.6)	0	0	0
Ventricular premature beat	1 (5.6)	1 (5.6)	0	0	0
Vomiting	1 (5.6)	1 (5.6)	0	0	0
Vomitting	1 (5.6)	0	1 (5.6)	0	0
hyperuricemia	1 (5.6)	1 (5.6)	0	0	0

Abbreviations: AE, adverse event; ALP, alkaline phosphatase; ALT: alanine aminotransferase; AST, aspartate aminotransferase; CTCAE, common terminology criteria for adverse events; GGT, gamma‐glutamyl transferase; PLT, platelet; TRAE, treatment‐related adverse event; WBC, white blood cell.

**TABLE 3:** Summary Treatment‐Related Adverse Events by CTCAE Grade Nab‐Paclitaxel dosage(mg/m^2^): 200.

TRAE	Any Grade n (%)	Grade 1–2 n (%)	Grade 3 n (%)	Grade 4 n (%)	Grade 5 n (%)
Treatment‐Related AE	7 (77.8)	5 (55.6)	1 (11.1)	1 (11.1)	0
Anaemia	3 (33.3)	3 (33.3)	0	0	0
PLT count decreased	2 (22.2)	0	1 (11.1)	1 (11.1)	0
Sinus tachycardia	2 (22.2)	2 (22.2)	0	0	0
Skin itch	2 (22.2)	2 (22.2)	0	0	0
WBC count decreased	2 (22.2)	1 (11.1)	1 (11.1)	0	0
Abdominal distension	1 (11.1)	1 (11.1)	0	0	0
Bacteremia	1 (11.1)	0	1 (11.1)	0	0
CK elevation	1 (11.1)	1 (11.1)	0	0	0
Headache	1 (11.1)	1 (11.1)	0	0	0
Hypokalemia	1 (11.1)	1 (11.1)	0	0	0
Musculoskeletal pain	1 (11.1)	1 (11.1)	0	0	0
Night sweats	1 (11.1)	1 (11.1)	0	0	0
Rash	1 (11.1)	1 (11.1)	0	0	0
Urine glucose elevation	1 (11.1)	1 (11.1)	0	0	0

Abbreviations: AE, adverse event; CK, creatine kinase; CTCAE, common terminology criteria for adverse events; PLT, platelet; TRAE, treatment‐related adverse event.

Regarding irAEs, the overall incidence was 55.6% (15/27) for all grades and 22.2% (6/27) for grade 3–4 events (Table 4). The most common severe irAE was infection (7.4%), followed by rash (7.4%), along with isolated cardiac or hematologic events.

**TABLE 4:** Summary Immune‐Related Adverse Events by CTCAE Grade.

irAE	Any Grade n (%)	Grade 1–2 n (%)	Grade 3 n (%)	Grade 4 n (%)	Grade 5 n (%)
Treatment‐Related AE	15 (55.6)	9 (33.3)	5 (18.5)	1 (3.7)	0
Anaemia	7 (25.9)	6 (22.2)	1 (3.7)	0	0
Skin itch	5 (18.5)	5 (18.5)	0	0	0
Rash	3 (11.1)	2 (7.4)	1 (3.7)	0	0
ALT elevation	2 (7.4)	2 (7.4)	0	0	0
AST elevation	2 (7.4)	2 (7.4)	0	0	0
Abdominal distension	2 (7.4)	2 (7.4)	0	0	0
Infection	2 (7.4)	0	2 (7.4)	0	0
Sinus tachycardia	2 (7.4)	2 (7.4)	0	0	0
Acute heart failure	1 (3.7)	0	0	1 (3.7)	0
Arrhythmia	1 (3.7)	0	0	1 (3.7)	0
Belching	1 (3.7)	1 (3.7)	0	0	0
Bone marrow suppression	1 (3.7)	0	0	1 (3.7)	0
CK elevation	1 (3.7)	1 (3.7)	0	0	0
Constipation	1 (3.7)	1 (3.7)	0	0	0
Dizziness	1 (3.7)	1 (3.7)	0	0	0
Fatigue	1 (3.7)	0	1 (3.7)	0	0
Gastrooesophageal burning	1 (3.7)	1 (3.7)	0	0	0
Hypoadrenal function	1 (3.7)	0	1 (3.7)	0	0
Musculoskeletal pain	1 (3.7)	1 (3.7)	0	0	0
Rhinitis	1 (3.7)	1 (3.7)	0	0	0
Total bilirubin elevation	1 (3.7)	1 (3.7)	0	0	0
Urine glucose elevation	1 (3.7)	1 (3.7)	0	0	0
WBC count decreased	1 (3.7)	1 (3.7)	0	0	0

*Note*: Each patient was counted once for the highest grade of each event experienced.

Abbreviations: AE, adverse event; ALT: alanine aminotransferase; AST, aspartate aminotransferase; CK, creatine kinase; CTCAE, common terminology criteria for adverse events; irAE, immune‐related adverse event; WBC, white blood cell.

### 2.4 Representative Case: Treatment Response and Pathological Changes

Focusing on patients who achieved surgical conversion after therapy, this study aimed to characterize the pathological basis for treatment success. Two patients achieved tumor regression sufficient for radical resection, both maintaining satisfactory treatment tolerance. One representative case involved cervical melanoma (histologically confirmed) showing PR after four cycles of DNV3 combination therapy (toripalimab, nab‐paclitaxel, cisplatin). Substantial tumor reduction was observed (Figure S1A: pretreatment; Figure S1B: posttreatment). Postoperative histopathology demonstrated degenerative melanoma cells with markedly diminished pigmentation (Figure S1C). And pathological examination of surgical specimens from two patients revealed marked remodeling of the tumor microenvironment following chemoimmunotherapy, with distinct patterns between individuals. One specimen exhibited dense immune cell infiltration occupying nearly 80% of the tumor area, with the remainder composed predominantly of fibrotic tissue (Figures S2A, S2B). In contrast, the second specimen showed a more balanced distribution, with immune infiltration and fibrosis each accounting for approximately half of the tumor region (Figures S2C, S2D). Pathological examination of the resected tissue confirmed not only degenerative tumor changes with reduced pigmentation but also a remodeled tumor microenvironment, underscoring the dual anti‐tumor activity of this combination strategy.

## 3 Discussion

This study proposes a new treatment regimen that combines DNV3 and toripalimab with chemotherapy for patients with advanced melanoma. Notably, this approach achieved an ORR of 54.4% among patients with liver metastases. Among the 11 patients with liver metastases, five treatment‐naïve individuals with mucosal melanoma subtypes experienced significant benefits from this combination therapy. Additionally, after optimizing the chemotherapy dosage, only 22.2% of patients experienced grade 3 or higher TRAEs. In this study, no biomarkers associated with treatment response were identified.

The efficacy observed in refractory melanoma constitutes a notable therapeutic advancement. Current second‐line treatments for PD‐(L)1–resistant disease remain unsatisfactory: nivolumab–ipilimumab achieves an approximate 30% ORR but is accompanied by substantial toxicity (grade 3–4 AEs: 55%) [7]; relatlimab–nivolumab, while better tolerated, yields only 11.7% ORR in the RELATIVITY‐020 trial [16]. Chemoimmunotherapy regimens combining PD‐1 inhibitors with taxanes demonstrate modest efficacy (ORR: 20–25%) [15]. By contrast, the quadruplet regimen nearly doubled the response rate (42.9%) while maintaining acceptable tolerability after dose adjustment. The median PFS of 7.36 months in PD‐(L)1–resistant cases further indicated its therapeutic promise, as most salvage options such as ipilimumab or targeted therapies generally yield PFS of only 2–4 months in comparable populations [17, 18, 19]. These results suggest that concurrent LAG‐3/PD‐1 inhibition combined with chemotherapy may effectively overcome primary resistance pathways in melanoma, although further mechanistic studies are warranted.

Distinct response profiles among melanoma subtypes yield clinically relevant insights. The regimen achieved 50% ORR in treatment‐naïve mucosal melanoma and 54.5% ORR in hepatic metastases—substantially exceeding historical anti–PD‐1 monotherapy outcomes (0–13.3% for mucosal melanoma [20, 21]; 4.3–8.5% for hepatic metastases) [16, 22]. The efficacy also surpasses that of nivolumab/relatlimab (43.1% ORR in unselected melanoma) [6], suggesting that the quadruplet regimen may overcome two central resistance mechanisms: intrinsic immunotherapy refractoriness in mucosal, and the immunosuppressive milieu characteristic of hepatic metastases. The 54.5% response rate in liver lesions represents a meaningful therapeutic improvement for this poor‐prognosis cohort.

The key registration trials CheckMate‐067 (nivolumab–ipilimumab) and RELATIVITY‐047 (nivolumab–relatlimab) primarily enrolled patients with cutaneous melanoma, while mucosal and acral subtypes collectively constituted less than 5% of participants. In contrast, these subtypes represent over 60% of melanoma cases in China. Their distinct tumor immune microenvironment—characterized by lower tumor mutational burden, diminished CD8^+^ T‐cell infiltration, and elevated VEGF expression—restricts the applicability of outcomes from international trials to Chinese patients. To improve contextual relevance, an indirect comparison with Chinese real‐world studies is presented in Supplementary Table 11, alongside clinical data from our center. In this advanced Chinese melanoma cohort, enriched for mucosal and acral subtypes, the combination regimen demonstrated a notable trend toward improved objective response rate relative to outcomes reported for existing immunotherapies in comparable populations. Despite potential discrepancies in adverse event reporting across studies, the incidence of Grade ≥3 TRAEs with this regimen remained consistent with the overall safety profile of nivolumab‐relatlimab in RELATIVITY‐047 and was markedly lower than that of nivolumab‐ipilimumab, indicating acceptable tolerability in clinical settings. These supplementary analyses contextualize the efficacy and safety of the combination regimen within population‐specific heterogeneity.

The selection of nab‐paclitaxel combined with cisplatin as the chemotherapy backbone was based on established clinical evidence and practical considerations in melanoma management. This regimen leverages the well‐recognized synergistic interaction between platinum compounds and taxanes, which has demonstrated enhanced antitumor efficacy in advanced melanoma while maintaining a predictable and tolerable safety profile [23]. From a clinical standpoint, nab‐paclitaxel confers distinct advantages over solvent‐based paclitaxel, including the elimination of premedication for hypersensitivity and a generally more favorable toxicity profile—an attribute particularly relevant in combination regimens [24]. Evidence from phase II trials indicates that nab‐paclitaxel provides efficacy comparable to dacarbazine with a distinct and manageable toxicity spectrum. The inclusion of cisplatin is further supported by its established role in melanoma, where cisplatin‐based combinations have yielded response rates of 15–20% in chemotherapy‐naïve metastatic disease [15]. Together, this chemotherapy backbone forms a clinically validated foundation for integration with immune checkpoint inhibitors [25], balancing efficacy and tolerability—a consideration that gains further relevance in light of the pathological changes observed in patient samples.

The absence of a clear association between BRAF or PD‐L1 status and treatment response implies potential broad applicability of this regimen; however, the limited sample size precludes firm conclusions. Among the enrolled patients, four carried BRAF mutations and twenty‐three were wild‐type. The observed BRAF V600E mutation frequency of 17.4% aligns with data from multicenter melanoma registries in China, indicating that the cohort reflects the expected mutational distribution within the regional population. Although a trend toward higher response was observed in BRAF‐mutated cases, comparison between four and twenty‐three patients lacks statistical robustness. BRAF V600E melanoma is characterized by constitutive MAPK pathway activation and an immunosuppressive microenvironment [26], typically involving diminished CD8^+^ T‐cell infiltration and elevated VEGF/PD‐L1 expression [27]. Such tumors generally respond poorly to monotherapy with immune checkpoint inhibitors, with reported response rates around 20–25% [28]. The chemoimmunotherapy regimen applied in this study may exert enhanced efficacy through complementary mechanisms: paclitaxel‐based therapy promotes tumor antigen release and modulates Treg activity, enhancing checkpoint inhibition, whereas cisplatin‐induced DNA damage increases neoantigen generation. Collectively, these effects may potentiate immune responsiveness in tumors harboring distinct genomic profiles such as BRAF V600E mutation. Cutaneous melanoma patients, particularly those with PD‐L1 CPS ≥5, achieved a 100% ORR, suggesting enhanced susceptibility to this combination. In contrast, the modest responses in mucosal and acral melanomas even with positive PD‐L1 expression reinforce the rationale for combining chemotherapy with immunotherapy to remodel the tumor microenvironment in these resistant subtypes. Consequently, The potential interaction between genomic alterations and treatment response merits investigation in larger cohorts.

The finding that all objective responders with liver metastases were treatment‐naïve mucosal melanoma patients indicates that this specific subset—treatment‐naïve mucosal melanoma with hepatic involvement—may derive particular benefit from the combination regimen. This contrasts with the lack of response in previously treated mucosal melanoma patients with liver metastases, aligning with the overall lower response rate observed in the pretreated mucosal subgroup. The 50% ORR in the small cohort of pretreated, non‐mucosal liver metastases suggests a differential treatment effect based on both tumor subtype and prior therapy. Given the exploratory nature of these findings, future prospective studies should prioritize stratified enrollment of treatment‐naïve mucosal melanoma patients with hepatic metastases to confirm the therapeutic relevance of this regimen within this biologically distinct population.

The safety profile of the regimen was rendered manageable through proactive dose adjustment. The dose modification for nab‐paclitaxel, from 260 mg/m^2^ to 200 mg/m^2^, was primarily driven by the emergence of Grade 3–5 myelosuppression in the initial cohort. Importantly, this reduction did not lead to a loss of therapeutic activity, as evidenced by comparable median PFS between the two cohorts. Considering the maintained antitumor efficacy and improved tolerability, 200 mg/m^2^ was designated as the Recommended Phase II Dose (RP2D) for nab‐paclitaxel in this combination regimen and adopted for subsequent clinical investigations. This dose adjustment strategy effectively minimized clinically significant hematologic toxicity while preserving the ability to assess preliminary efficacy. The observed spectrum of irAEs aligned with the established toxicity pattern of LAG‐3/PD‐1 blockade, and no novel safety concerns emerged relative to the known profile of this immunotherapy combination.

The primary limitations of this study include its small sample size and single‐arm design, which preclude definitive statistical comparisons and firm conclusions regarding biomarker associations. Despite these constraints, we have observed potential differences in treatment response among histological subtypes—particularly in mucosal melanoma and patients with liver metastases. Consequently, we have formally incorporated histological subtype as a stratification factor in our subsequent national multicenter Investigational New Drug (IND) registration trial (ClinicalTrials.gov ID: [CXSL2400526]). This prospective validation is critical to determine whether distinct pathological phenotypes correlate with clinical outcomes. Furthermore, the sample size of 27 participants limits the statistical power to detect rare adverse events, particularly those with an estimated incidence in the range of 1–5%. It is important to note that one of the primary aims of this work was to establish a preliminary safety profile to inform the design and feasibility of subsequent larger‐scale trials. Consequently, the assessment of rare adverse events requires further validation in future studies with expanded cohorts. Third, the heterogeneous TME remodeling observed pathologically underscores the complexity of individual responses. Future studies should, therefore, integrate multi‐omics approaches to elucidate the mechanistic basis for these differential responses and identify robust predictive biomarkers.

## 4 Conclusions

In summary, DNV3/toripalimab combined with chemotherapy exhibits meaningful therapeutic activity in refractory and mucosal melanoma, accompanied by a tolerable safety profile following dose optimization. The observed outcomes warrant continued investigation of this regimen in larger, randomized studies, with particular attention to Asian cohorts and underrepresented melanoma subtypes.

### 4.1 Patients and Methods

#### 4.1.1 Study Design

This single‐arm, prospective, open‐label, investigator‐initiated trial assessed the efficacy and safety of DNV3 combined with toripalimab and chemotherapy in patients with advanced melanoma. The study was conducted at Fujian Cancer Hospital between January 2024 and April 2025 in compliance with the ethical standards of the Declaration of Helsinki. Written informed consent was obtained from all enrolled patients. The trial was registered in the Chinese Clinical Trial Registry (ID: ChiCTR2400079543, www.chictr.org.cn). Given the lack of prior publicly available clinical data for this specific combination, conventional statistical power calculations based on historical effect sizes were not feasible. Therefore, as an exploratory Phase I/II investigation, this study employed a fixed‐sample‐size design with a predefined target of 20–40 evaluable patients.

#### 4.1.2 Patients

Eligible participants were aged ≥ 18 years with histologically confirmed unresectable or metastatic melanoma. Inclusion criteria comprised patients who were either treatment‐naïve or had experienced disease progression following at least one prior anti‐PD‐1–based regimen; patients who relapsed within six months after completing adjuvant or neoadjuvant anti‐PD‐1 therapy; and BRAF‐mutant patients who had progressed after at least one prior BRAF inhibitor regimen. Additional requirements included an Eastern ECOG performance status of 0 or 1 and the presence of at least one measurable extracranial lesion per RECIST v1.1, without previous irradiation to that lesion. Central nervous system (CNS) metastases were required to be asymptomatic and radiologically stable for at least three months. Participants were also required to have adequate hematologic and organ function and to provide consent for biopsy sampling. Exclusion criteria included a history of autoimmune disorders, active infections, or prior exposure to anti‐LAG‐3 therapy. In this study, ‘anti‐PD‐(L)1‐refractory patients’ were defined as those who had previously received anti‐PD‐(L)1 monotherapy or combination therapy and demonstrated radiologic disease progression per RECIST v1.1 during treatment or within 12 weeks after the final dose. All progression events underwent independent review by at least one radiologist and one clinical investigator to rule out pseudoprogression.

#### 4.1.3 Dosing

Chemotherapy and DNV3 dosages were calculated according to body surface area (m^2^) and weight (kg), whereas toripalimab was administered at a fixed dose. Each treatment cycle lasted 21 days, with combination therapy repeated every three weeks. On Day 1 of each cycle, chemotherapy agents such as nab‐paclitaxel and cisplatin were infused intravenously prior to DNV3 and toripalimab administration. Cisplatin was delivered from Day 1 to Day 3 in each cycle. The dosing schedule was as follows: DNV3, 3 mg/kg on Day 1 every 3 weeks; toripalimab, 240 mg fixed dose on Day 1 every 3 weeks; nab‐paclitaxel, 260 mg/m^2^ on Day 1; and cisplatin, 25 mg/m^2^ on Days 1–3 every 3 weeks for the initial six treatment cycles.

#### 4.1.4 Criteria for Dose Reduction and Treatment Discontinuation

Dose reduction was mandated upon the first incidence of any of the following: ≥ Grade 3 non‐hematologic toxicity; ≥ Grade 4 hematologic toxicity (including febrile neutropenia or bleeding‐related thrombocytopenia); or ≥ Grade 3 hematologic toxicity persisting for ≥7 days. Permanent discontinuation was required under the following conditions: any nab‐paclitaxel‐related serious adverse event, as defined by CTCAE v5.0; or recurrent ≥ Grade 3 toxicity deemed by the investigator to be unmanageable through dose adjustment or treatment interruption.

#### 4.1.5 Endpoints and Assessments

The primary endpoint was the ORR of the combined regimen comprising DNV3, toripalimab, and chemotherapy in patients with advanced melanoma. Secondary endpoints included safety, PFS, DOR, disease control rate (DCR) and OS. Tumor responses were assessed every six weeks according to RECIST v1.1 and iRECIST guidelines, with CR or PR requiring confirmation at least six weeks after initial documentation. Safety evaluation included the incidence of AEs, serious adverse events (SAEs), laboratory abnormalities, and treatment‐related fatalities. Monitoring of AEs continued throughout therapy and for 28 days following the final administration.

#### 4.1.6 PD‐L1 Expression Analysis in Tumour Biopsies

Pretreatment tumor biopsy specimens, either archival or newly obtained, were analyzed for PD‐L1 expression. Assessment was conducted via immunohistochemistry (IHC) using the SP142 antibody in a centralized laboratory. Certified pathologists determined PD‐L1 status, defining PD‐L1 positivity as membrane staining of ≥1% of tumor cells at any intensity.

### 4.2 Statistical Analysis

All statistical analyses were conducted with SAS (version 9.4). CIs for ORR and DCR were derived via the Clopper–Pearson method, whereas median values and corresponding CIs for DOR, PFS, and OS were obtained using the Kaplan–Meier approach. Adverse events, including SAEs, TRAEs, and treatment‐related SAEs, were graded for severity according to CTCAE v5.0. Baseline characteristics and safety outcomes were summarized using descriptive statistics, including mean, standard deviation, and median.

## Author Contributions

Financial support: Yu Chen, Jing Lin and Lizhu Chen. Administrative support: Yu Chen and Jing Lin. Provision of study material or patients: all authors. Collection and assembly of data: Lizhu Chen, Ling Chen, Dingyi Wang, Yuping Lu, Huishan Zhang, Ping Chen, Wei Yan, and Zuoxiang Xiao. Data analysis and interpretation: all authors. Manuscript writing: Jing Lin and Lizhu Chen. Final approval of manuscript: all authors. Accountable for all aspects of the work: all authors. All authors have read and approved the final manuscript.

## Funding Information

The study was sponsored by Zhejiang Shimai Pharmaceutical Co., Ltd., which provided the primary financial support for the clinical trial conduct, including investigational product supply and patient‐related costs. The government grants section supported peripheral aspects of the research, such as personnel training, laboratory analyses, and open‐access publication fees (Article Processing Charges, APCs). The government grants includes: The Natural Science Foundation of Fujian Province, China (Grant No. 2023J011254); Joint Funds for the Innovation of Science and Technology, Fujian province (Grant No. 2024Y9616, 2023Y9412); Young and Middle‐aged Scientific Research Major Project of Fujian Provincial Health Commission (Grant No. 2022ZQNZD009; 2025GGB029); the Special Research Funds for Local Science and Technology Development Guided by Central Government (Grant No. 2023L3020); National Natural Science Foundation of China (Grant No. 82350126).

## Conflicts of Interest

This study was supported by Zhejiang Shimai Pharmaceutical Co., Ltd. Author Zuoxiang Xiao is an employee in Zhejiang Shimai Pharmaceutical Co., but has no potential relevant financial or non‐financial interests to disclose. The other authors have no conflicts of interest to declare.

## Ethics Approval

The study protocol was approved by the Ethics Committee of Fujian Cancer Hospital (APPROVAL NUMBER: K2023‐446) and was conducted in accordance with the ethical standards of the Declaration of Helsinki. Informed consent was obtained from all participants in the study.

## Supporting information




**Figure S1**. Therapeutic response in cervical melanoma. (A) Baseline contrast‐enhanced CT (portal venous phase, 5 mm slice thickness) depicting the primary cervical lesion in axial view (red circle: maximal diameter 7.27 cm; mean density 45 HU). (B) Post‐treatment CT obtained using the same imaging protocol demonstrates a partial response (RECIST v1.1), with a 48% decrease in target lesion diameter (red circle: 3.74 cm). (C) Histopathological examination of the surgical specimen. Upper panel: H&E staining at 100×magnification (scale bar: 200 µm) reveals widespread degeneration of melanoma cells. Lower panel: High‐power field (400×, scale bar: 50 µm) shows residual intracellular pigment within degenerated tumor cells. CT, Computed Tomography; mm, millimeter; um, micrometer; cm, centimeter; RECIST, Response Evaluation Criteria in Solid Tumors; H&E, Hematoxylin and Eosin. **Figure S2**. Pathological assessment of tumor microenvironment remodeling in two representative patients after chemoimmunotherapy. (A, B) Pathological specimen from Patient 1 shows a response pattern dominated by dense immune cell infiltration, occupying approximately 80% of the tumor area, with the remainder composed predominantly of fibrotic tissue. (C, D) Specimen from Patient 2 displays a balanced pattern, with immune infiltration and fibrosis each accounting for about 50% of the tumor region. **Table S1**. ORR and DCR based on RECIST 1.1(Prior anti–PD‐(L)1 Therapy/Untreated Mucosal Subtype). **Table S2**. ORR and Disease Control Rate  DCR based on RECIST 1.1 Prior anti–PD‐(L)1 Therapy by Melanoma Subtype). **Table S3**.Progression Free Survival (PFS). **Table S4**.Progression Free Survival(Prior anti–PD‐(L)1 Therapy/Untreated Mucosal Subtype). **Table S5**.Progression Free Survival (Prior anti–PD‐(L)1 Therapy by Melanoma subtype). **Table S6**. Survival Rate. **Table S7**.Duration of Response(Prior anti–PD‐(L)1 Therapy/Untreated Mucosal Subtype). **Table S8**.Duration of Response(Prior anti–PD‐(L)1 Therapy by Melanoma subtype). **Table S9**. ORR and PFS based on RECIST 1.1 in 13 mucosal subtype. **Table S10**. ORR based on RECIST 1.1 in subtype with liver metastases. **Table S11**. ORR and DCR based on RECIST 1.1 with DNV3 plus toripalimab therapy (Prior anti–PD‐(L)1 Therapy). **Table S12**. PD‐L1 (CPS ≥1) expression analysis by IHC and its correlation with treatment response. **Table S13**. PD‐L1 (CPS ≥5) expression analysis by IHC and its correlation with treatment response.

## Data Availability

The datasets used and/or analysed during the current study are available from the corresponding author on reasonable request.
