# Supplementary material for: Efficacy and Safety of DNV3 (a Lymphocyte‐activation Gene 3–blocking Antibody) Combined With Toripalimab and Chemotherapy in Advanced Melanoma: An Open‐label, Single‐arm Clinical Trial
Source: MedComm (2020). 2026 Mar 2;7(3):e70648. doi: 10.1002/mco2.70648 (PMC12954137; doi:10.1002/mco2.70648)
Supplement: Supplementary file 1 — Figure S1. Therapeutic response in cervical melanoma. (A) Baseline contrast‐enhanced CT (portal venous phase, 5 mm slice thickness) depicting the primary cervical lesion in axial view (red circle: maximal diameter 7.27 cm; mean density 45 HU). (B) Post‐treatment CT obtained using the same imaging protocol demonstrates a partial response (RECIST v1.1), with a 48% decrease in target lesion diameter (red circle: 3.74 cm). (C) Histopathological examination of the surgical specimen. Upper panel: H&E staining at 100×magnification (scale bar: 200 µm) reveals widespread degeneration of melanoma cells. Lower panel: High‐power field (400×, scale bar: 50 µm) shows residual intracellular pigment within degenerated tumor cells. CT, Computed Tomography; mm, millimeter; um, micrometer; cm, centimeter; RECIST, Response Evaluation Criteria in Solid Tumors; H&E, Hematoxylin and Eosin. Figure S2. Pathological assessment of tumor microenvironment remodeling in two representative patients after chemoimmunotherapy. (A, B) Pathological specimen from Patient 1 shows a response pattern dominated by dense immune cell infiltration, occupying approximately 80% of the tumor area, with the remainder composed predominantly of fibrotic tissue. (C, D) Specimen from Patient 2 displays a balanced pattern, with immune infiltration and fibrosis each accounting for about 50% of the tumor region. Table S1. ORR and DCR based on RECIST 1.1(Prior anti–PD‐(L)1 Therapy/Untreated Mucosal Subtype). Table S2. ORR and Disease Control Rate DCR based on RECIST 1.1 Prior anti–PD‐(L)1 Therapy by Melanoma Subtype). Table S3.Progression Free Survival (PFS). Table S4.Progression Free Survival(Prior anti–PD‐(L)1 Therapy/Untreated Mucosal Subtype). Table S5.Progression Free Survival (Prior anti–PD‐(L)1 Therapy by Melanoma subtype). Table S6. Survival Rate. Table S7.Duration of Response(Prior anti–PD‐(L)1 Therapy/Untreated Mucosal Subtype). Table S8.Duration of Response(Prior anti–PD‐(L)1 Therapy by Melanoma subtype). Tabl [file MCO2-7-e70648-s001.docx]

**Title: Efficacy and safety of DNV3 combined with toripalimab and chemotherapy in advanced melanoma: an open-label, single-arm clinical trial**

Jing Lin^1, 2, 3#^, Lizhu Chen^1, 2#^, Ling Chen^1, 2, 3^, Dingyi Wang^3^, Yuping Lu^1, 2^, Huishan Zhang^3^, Ping Chen^1, 2^, Wei Yan^1, 2^, Zuoxiang Xiao^4^, Yu Chen^1, 2, 3*^

^1^ Department of Medical Oncology, Clinical Oncology School of Fujian Medical University, Fujian Cancer Hospital, Fuzhou, 350014, Fujian Province, China

^2^ Cancer Bio-Immunotherapy Center, Clinical Oncology School of Fujian Medical University, Fujian Cancer Hospital, Fuzhou, 350014, Fujian Province, China.

^3^ Department of Phase I Clinical Trial Ward, Clinical Oncology School of Fujian Medical University, Fujian Cancer Hospital, Fuzhou, 350014, Fujian Province, China.

^4^ Zhejiang Shimai Pharmaceutical Co., Ltd., Hangzhou, 321000, Zhejiang Province, China

**Correspondence:** Yu Chen [(chenyu1980@fjmu.edu.cn)](mailto:(docshijie@aliyun.com))

^#^These authors contributed equally: Jing Lin, Lizhu Chen

**Supplementary Figures**

**
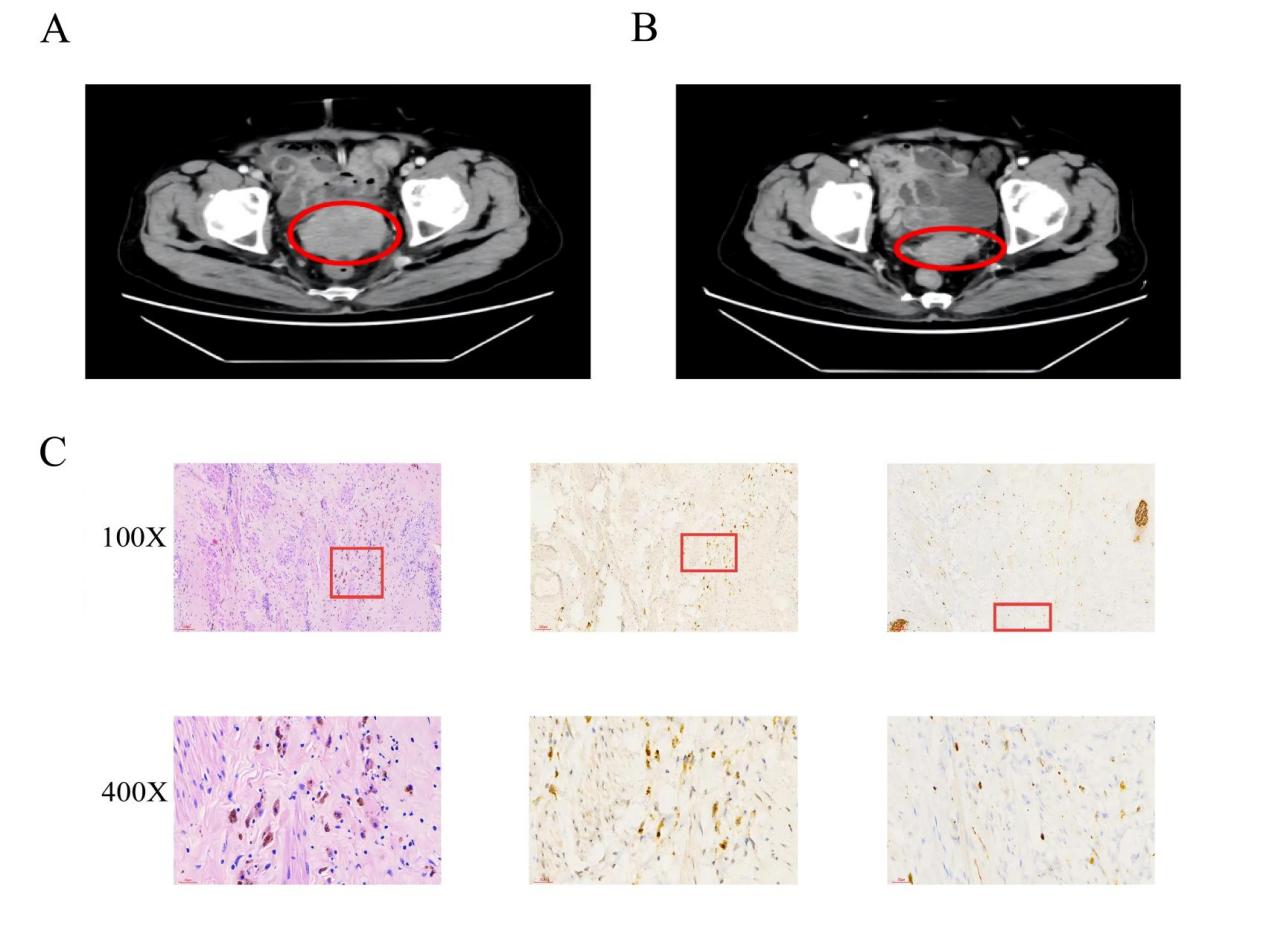
**

**Figure S1**. Therapeutic response in cervical melanoma. (A) Baseline contrast-enhanced CT (portal venous phase, 5 mm slice thickness) depicting the primary cervical lesion in axial view (red circle: maximal diameter 7.27 cm; mean density 45 HU). (B) Post-treatment CT obtained using the same imaging protocol demonstrates a partial response (RECIST v1.1), with a 48% decrease in target lesion diameter (red circle: 3.74 cm). (C) Histopathological examination of the surgical specimen. Upper panel: H&E staining at 100×magnification (scale bar: 200 μm) reveals widespread degeneration of melanoma cells. Lower panel: High-power field (400×, scale bar: 50 μm) shows residual intracellular pigment within degenerated tumor cells. CT, Computed Tomography; mm, millimeter; um, micrometer; cm, centimeter; RECIST, Response Evaluation Criteria in Solid Tumors; H&E, Hematoxylin and Eosin.


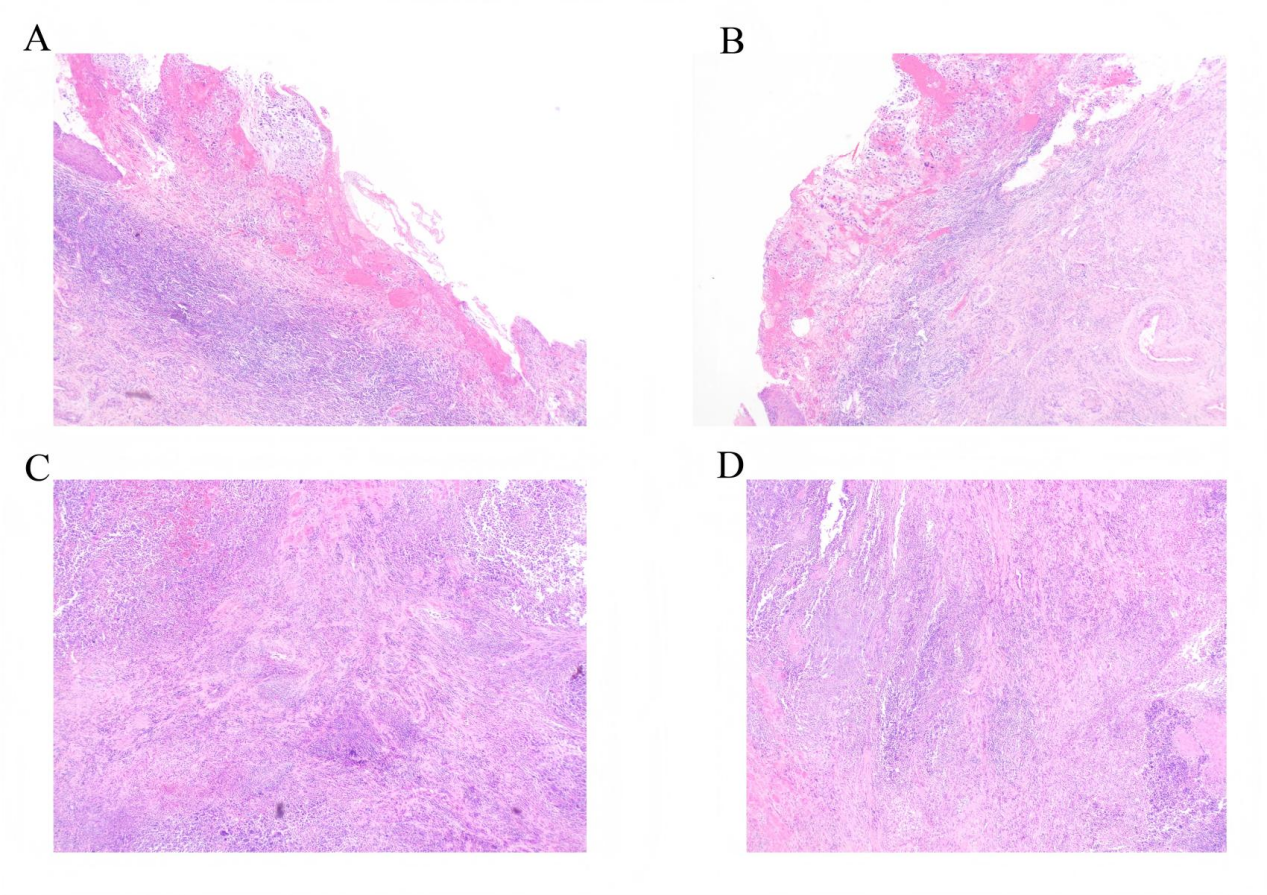


**Figure S2.** Pathological assessment of tumor microenvironment remodeling in two representative patients after chemoimmunotherapy. (A, B) Pathological specimen from Patient 1 shows a response pattern dominated by dense immune cell infiltration, occupying approximately 80% of the tumor area, with the remainder composed predominantly of fibrotic tissue. (C, D) Specimen from Patient 2 displays a balanced pattern, with immune infiltration and fibrosis each accounting for about 50% of the tumor region.

**Supplementary tables**

| **Table S1 ORR and DCR based on RECIST 1.1（Prior anti–PD-(L)1 Therapy/Untreated Mucosal Subtype）** | | |
| --- | --- | --- |
| **Characteristics** | **Patients with Prior anti–PD-(L)1 Therapy (N = 21)** | **Patients of Untreated Mucosal Subtype (N = 6)** |
|  | | |
| Best overall response  CR | 2 (9.5) | 0 |
| PR | 7 (33.3) | 3 (50.0) |
| uPR | 0 | 0 |
| SD | 9 (42.9) | 2 (33.3) |
| PD | 2 (9.5) | 0 |
| NE | 1 (4.8) | 1 (16.7) |
|  | | |
| ORR | 42.9 | 50.0 |
| (95% CI) | (21.8, 66.0) | (11.8, 88.2) |
|  | | |
| DCR | 85.7 | 83.3 |
| (95% CI) | (63.7, 97.0) | (35.9, 99.6) |
|  | | |
| Note: N = number of subjects in the analysis population, Nx = number of subjects with non-missing value, n = number of subjects in the specific category, percentage (%) = (n/N)*100. CI = confidence interval. ORR = CR + PR. DCR= CR + PR + SD + uPR. TheCI is calculated by Clopper-Pearson method.  Abbreviations: ORR, objective response rate; DCR, disease control rate; RECIST, response evaluation criteria in solid tumors; PD-(L)1, programmed death -(ligand)1; CR, complete response; PR, partial response; uPR, unconfirmed partial response; SD, stable disease; PD, progressive disease; NE, not evaluable; CI, confidence interval. | | |

| **Table S2 ORR and Disease Control Rate  DCR based on RECIST 1.1 Prior anti–PD-(L)1 Therapy by Melanoma Subtype）** | | | | |
| --- | --- | --- | --- | --- |
| **Characteristics** | **Mucosal (N = 7)** | **Cutaneous (N = 5)** | **Acral (N = 6)** | **Unknown (N = 3)** |
|  | | | | |
| Best overall response  CR | 2 (28.6) | 0 | 0 | 0 |
| PR | 0 | 5 (100) | 1 (16.7) | 1 (33.3) |
| uPR | 0 | 0 | 0 | 0 |
| SD | 3 (42.9) | 0 | 5 (83.3) | 1 (33.3) |
| PD | 2 (28.6) | 0 | 0 | 0 |
| NE | 0 | 0 | 0 | 1 (33.3) |
|  | | | | |
| ORR | 28.6 | 100.0 | 16.7 | 33.3 |
| (95% CI) | (3.7, 71.0) | (47.8, 100.0) | (0.4, 64.1) | (0.8, 90.6) |
|  | | | | |
| DCR | 71.4 | 100.0 | 100.0 | 66.7 |
| (95% CI) | (29.0, 96.3) | (47.8, 100.0) | (54.1, 100.0) | (9.4, 99.2) |
|  | | | | |
| Note: N = number of subjects in the analysis population, Nx = number of subjects with non-missing value, n = number of subjects in the specific category, percentage (%) = (n/N)*100. CI = confidence interval. ORR = CR + PR. DCR= CR + PR + SD + uPR. The CI is calculated by Clopper-Pearson method.  Abbreviations: ORR, objective response rate; DCR, disease control rate; RECIST, response evaluation criteria in solid tumors; PD-(L)1, programmed death -(Ligand)1; CR, complete response; PR, partial response; uPR, unconfirmed partial pesponse; SD, stable disease; PD, progressive disease; NE, not evaluable; CI, confidence interval. | | | | |

| **Table S3 Progression Free Survival (PFS)** | | |
| --- | --- | --- |
|  | **Total (N = 27)** | **Patients with liver metastases**  **(N = 11)** |
|  | | |
| Status  Disease progression | 13 (48.1) |  |
| Death | 2 (7.4) |  |
| Censoring | 12 (44.4) | 3(27.2) |
|  |  |  |
| Median of PFS (month) | 7.29 | 4.96 |
| (95% CI) | (4.96, 10.28) | (1.38, -) |
|  | | |
| Note: N = number of subjects in the analysis population, Nx = number of subjects with non-missing value, n = number of subjects in the specific category, percentage (%) = (n/N)*100.  The median  and CI of PFS is estimated by Kaplan-Meier method.  Abbreviations: CI, confidence interval; PFS, progression free survival.   Page 1 of 1 | | |

| **Table S4 Progression Free Survival（Prior anti–PD-(L)1 Therapy/Untreated Mucosal Subtype）** | | |
| --- | --- | --- |
|  | **Patients with Prior anti–PD-(L)1 Therapy (N = 21)** | **Patients of Untreated Mucosal Subtype (N = 6)** |
|  | | |
| Status  Disease progression | 10 (47.6) | 3 (50.0) |
| Death | 2 (9.5) | 0 |
| Censoring | 9 (42.9) | 3 (50.0) |
|  | | |
| Median of PFS (month) | 7.36 | 5.78 |
| (95% CI) | (4.96, 10.28) | (2.83, -) |
|  | | |
| Note: N = number of subjects in the analysis population, Nx = number of subjects with non-missing value, n = number of subjects in the specific category, percentage (%) = (n/N)*100.  The median and CI of PFS is estimated by Kaplan-Meier method.  Abbreviations: CI, confidence interval; PFS, progression free survival; PD-(L)1, programmed death -(ligand)1.   Page 1 of 1 | | |

| **Table S5 Progression Free Survival （Prior anti–PD-(L)1 Therapy by Melanoma subtype）** | | | | |
| --- | --- | --- | --- | --- |
|  | **Mucosal (N = 7)** | **Cutaneous (N = 5)** | **Acral (N = 6)** | **Unknown (N = 3)** |
|  | | | | |
| Status  Disease progression | 4 (57.1) | 1 (20.0) | 4 (66.7) | 1 (33.3) |
| Death | 0 | 0 | 1 (16.7) | 1 (33.3) |
| Censoring | 3 (42.9) | 4 (80.0) | 1 (16.7) | 1 (33.3) |
|  | | | | |
| Median of PFS (month) | 8.44 | - | 6.14 | 5.17 |
| (95% CI) | (1.31, -) | (10.28, -) | (2.63, -) | (2.99, -) |
|  | | | | |
| Note: N = number of subjects in the analysis population, Nx = number of subjects with non-missing value, n = number of subjects in the specific category, percentage (%) = (n/N)*100.  The median and CI of PFS is estimated by Kaplan-Meier method.  Abbreviations: CI, confidence interval; PFS, progression free survival; PD-(L)1, programmed death -(ligand)1.   Page 1 of 1 | | | | |

| **Table S6 Survival Rate** | | | | |
| --- | --- | --- | --- | --- |
|  | **Total (N = 27)** | **Prior anti-PD-(L)1 therapy (N = 21)** | **Patients of Untreated Mucosal Subtype (N = 6)** | **Mucosal (N = 13)** |
|  | | | | |
| PFS survival rate at 6 months | 60.0 | 68.1 | 0.0 | 40.3 |
| (95% CI) | (36.4, 77.2) | (42.0, 84.4) | (-, -) | (10.7, 69.1) |
|  | | | | |
| PFS survival rate at 9 months | 30.3 | 34.4 | 0.0 | 26.9 |
| (95% CI) | (11.6, 51.6) | (13.1, 57.1) | (-, -) | (4.3, 57.6) |
|  | | | | |
| PFS survival rate at 12 months | 15.1 | 17.2 | 0.0 | - |
| (95% CI) | (1.4, 43.7) | (1.4, 48.1) | (-, -) | (-, -) |
|  | | | | |
| OS survival rate at 6 months | 95.8 | 94.7 | 100.0 | 100.0 |
| (95% CI) | (73.9, 99.4) | (68.1, 99.2) | (100.0, 100.0) | (100.0, 100.0) |
|  | | | | |
| OS survival rate at 9 months | 87.8 | 85.3 | 100.0 | 100.0 |
| (95% CI) | (57.1, 97.1) | (50.5, 96.4) | (100.0, 100.0) | (100.0, 100.0) |
|  | | | | |
| OS survival rate at 12 months | 87.8 | 85.3 | 100.0 | 100.0 |
| (95% CI) | (57.1, 97.1) | (50.5, 96.4) | (100.0, 100.0) | (100.0, 100.0) |
|  | | | | |
| Note: N = number of subjects in the analysis population, Nx = number of subjects with non-missing value, n = number of subjects in the specific category, percentage(%) = (n/N)*100.  The survival rate and CI of PFS/OS is estimated by Kaplan-Meier method.  Abbreviations: CI, confidence interval; PFS, progression free survival; PD-(L)1, programmed death -(ligand)1; OS, overall survival. | | | | |

| **Table S7 Duration of Response（Prior anti–PD-(L)1 Therapy/Untreated Mucosal Subtype）** | | |
| --- | --- | --- |
|  | **Patients with Prior anti–PD-(L)1 Therapy (N = 21)** | **Patients of Untreated Mucosal Subtype (N = 6)** |
|  | | |
| Subjects with confirmed CR or PR | 9 (42.9) | 3 (50.0) |
| Disease progression | 2 (9.5) | 1 (16.7) |
| Death | 1 (4.8) | 0 |
| Censoring | 6 (28.6) | 2 (33.3) |
|  | | |
| Median of DoR (month) | 8.67 | 4.57 |
| (95% CI) | (3.65, -) | (-, -) |
|  | | |
| Note: N = number of subjects in the analysis population, Nx = number of subjects with non-missing value, n = number of subjects in the specific category, percentage (%) = (n/N)*100.  The median and CI of DoR is calculated by Kaplan-Meier method.  Abbreviations: CI, confidence interval; PD-(L)1, programmed death-(ligand)1; DoR, duration of response; CR, complete response; PR, partial response. | | |

| **Table S8 Duration of Response（Prior anti–PD-(L)1 Therapy by Melanoma subtype）** | | | | |
| --- | --- | --- | --- | --- |
|  | **Mucosal (N = 7)** | **Cutaneous (N = 5)** | **Acral (N = 6)** | **Unknown (N = 3)** |
|  | | | | |
| Subjects with confirmed CR or PR | 2 (28.6) | 5 (100) | 1 (16.7) | 1 (33.3) |
| Disease progression | 0 | 1 (20.0) | 1 (16.7) | 0 |
| Death | 0 | 0 | 0 | 1 (33.3) |
| Censoring | 2 (28.6) | 4 (80.0) | 0 | 0 |
|  | | | | |
| Median of DoR (month) | - | - | 3.65 | 4.47 |
| (95% CI) | (-, -) | (8.67, -) | (-, -) | (-, -) |
|  | | | | |
| Note: N = number of subjects in the analysis population, Nx = number of subjects with non-missing value, n = number of subjects in the specific category, percentage (%) = (n/N)*100.  The median and CI of DoR is calculated by Kaplan-Meier method.  Abbreviations: CI, confidence interval; PD-(L)1, programmed death-(ligand)1; DoR, duration of response; CR, complete response; PR, partial response. | | | | |

| **Table S9 ORR and PFS based on RECIST 1.1 in 13 mucosal subtype** | | |
| --- | --- | --- |
|  | **Prior anti-PD-(L)1 therapy (N = 7)** | **Patients of Untreated l Subtype (N = 6)** |
|  | | |
| Median of PFS (month) | 8.44 | 5.78 |
| (95% CI) | (1.31, -) | (2.83, -) |
|  | | |
| Overall response rate (ORR) | 28.6 | 50.0 |
| (95% CI) | (3.7, 71.0) | (11.8, 88.2) |
|  | | |
| Note: N = number of subjects in the analysis population  The median and CI of PFS is estimated by Kaplan-Meier method.  Abbreviations: CI, confidence interval; PD-(L)1, programmed death-(ligand)1; PFS, progression free survival; ORR, overall response rate. | | |

| **Table S10 ORR based on RECIST 1.1 in subtype with liver metastases** | | | |
| --- | --- | --- | --- |
| **Characteristics** | | **PR/CR (N)** | **Total**  **(N)** |
|  | | | |
| **Mucosal** | **Mucosal (total)** | 3 | 7 |
|  | **Untreated Mucosal Subtype** | 3 | 5 |
|  | **Prior anti–PD-(L)1 Therapy** | 0 | 2 |
| **Cutaneous** | | 1 | 1 |
| **Acral** | | 2 | 2 |
| **Unknown** | | 1 | 1 |
| **Prior anti–PD-(L)1 Therapy** | | 4 | 6 |
|  | | | |
| Note: N = number of subjects in the analysis population. ORR = CR + PR.  Abbreviations: PD-(L)1, programmed death-(ligand)1; CR, complete response; PR, partial response; ORR, overall response rate; RECIST, response evaluation criteria in solid tumors. | | | |

| **Table S11 ORR and DCR based on RECIST 1.1 with DNV3 plus toripalimab therapy（Prior anti–PD-(L)1 Therapy）** | | |
| --- | --- | --- |
| **Characteristics** | **Cutaneous (N = 10)** | **Acral**  **(N = 14)** |
|  | | |
| Overall response rate (ORR) | 10.0 | 21.4 |
|  | | |
| Disease control rate (DCR) | 60.0 | 57.1 |
|  | | |
| Note: N = number of subjects in the analysis population, n = number of subjects in the specific category, percentage (%) = (n/N)*100.  Abbreviations: ORR, overall response rate; DCR, disease control rate; PD-(L)1, programmed death-(ligand)1; RECIST, response evaluation criteria in solid tumors. | | |

| **Table S12 PD-L1 (CPS ≥1) expression analysis by IHC and its correlation with treatment response** | | | |
| --- | --- | --- | --- |
| **Characteristics** | | **PR/CR (N)** | **Total**  **(N)** |
|  | | | |
| **Mucosal** | **Mucosal (total)** | 1 | 3 |
|  | **Untreated Mucosal Subtype** | 0 | 1 |
|  | **Prior anti–PD-(L)1 Therapy** | 1 | 2 |
| **Cutaneous** | | 4 | 4 |
| **Acral** | | 1 | 2 |
| **Unknown** | | 0 | 1 |
| **Prior anti–PD-(L)1 Therapy** | | 6 | 9 |
|  | | | |
| Note: N = number of subjects in the analysis population. Abbreviations: PD-(L)1, programmed death-(ligand)1; CPS, combined positive score; IHC, immunohistochemistry; CR, complete response; PR, partial response. | | | |

| **Table S13 PD-L1 (CPS ≥5) expression analysis by IHC and its correlation with treatment response** | | | |
| --- | --- | --- | --- |
| **Characteristics** | | **PR/CR (N)** | **Total**  **(N)** |
|  | | | |
| **Mucosal** | **Mucosal** | 0 | 2 |
|  | **Untreated Mucosal Subtype** | 0 | 1 |
|  | **Prior anti–PD-(L)1 Therapy** | 0 | 1 |
| **Cutaneous** | | 2 | 2 |
| **Acral** | | 0 | 0 |
| **Unknown** | | 1 | 1 |
| **Prior anti–PD-(L)1 Therapy** | | 3 | 4 |
|  | | | |
| Note: N = number of subjects in the analysis population. Abbreviations: PD-(L)1, programmed death-(ligand)1; CPS, combined positive score; IHC, immunohistochemistry; CR, complete response; PR, partial response. | | | |
